# Severe imported malaria in an intensive care unit: a review of 59 cases

**DOI:** 10.1186/1475-2875-11-96

**Published:** 2012-03-29

**Authors:** Lurdes C Santos, Cândida F Abreu, Sandra M Xerinda, Margarida Tavares, Raquel Lucas, António C Sarmento

**Affiliations:** 1 ICU Infectious Disease Unit, Hospital de S. João, Alameda do Professor Hernâni Monteiro, 4202-451 Porto, Portugal; 2 Infectious Disease Department, University of Porto Medical School. C. H. S. João, Porto, Portugal; 3 Department of Clinical Epidemiology, Predictive Medicine and Public Health, University of Porto Medical School, Porto, Portugal

**Keywords:** *Plasmodium falciparum*, Severe malaria, Prognosis, Shock, Multi-organic dysfunction, ARDS, ICU, Fatality rate

## Abstract

**Background:**

In view of the close relationship of Portugal with African countries, particularly former Portuguese colonies, the diagnosis of malaria is not a rare thing. When a traveller returns ill from endemic areas, malaria should be the number one suspect. World Health Organization treatment guidelines recommend that adults with severe malaria should be admitted to an intensive care unit (ICU).

**Methods:**

Severe cases of malaria in patients admitted to an ICU were reviewed retrospectively (1990-2011) and identification of variables associated with in-ICU mortality performed. Malaria prediction score (MPS), malaria score for adults (MSA), simplified acute physiology score (SAPSII) and a score based on WHO's malaria severe criteria were applied. Statistical analysis was performed using StataV12.

**Results:**

Fifty nine patients were included in the study, all but three were adults; 47 (79,6%) were male; parasitaemia on admission, quantified in 48/59 (81.3%) patients, was equal or greater than 2% in 47 of them (97.9%); the most common complications were thrombocytopaenia in 54 (91.5%) patients, associated with disseminated intravascular coagulation (DIC) in seven (11.8%), renal failure in 31 (52.5%) patients, 18 of which (30.5%) oliguric, shock in 29 (49.1%) patients, liver dysfunction in 27 (45.7%) patients, acidaemia in 23 (38.9%) patients, cerebral dysfunction in 22 (37.2%) patients, 11 of whom with unrousable coma, pulmonary oedema/ARDS in 22 (37.2%) patients, hypoglycaemia in 18 (30.5%) patients; 29 (49.1%) patients presented five or more dysfunctions. The case fatality rate was 15.2%. Comparing the four scores, the SAPS II and the WHO score were the most sensitive to death prediction. In the univariate analysis, death was associated with the SAPS II score, cerebral malaria, acute renal and respiratory failure, DIC, spontaneous bleeding, acidosis and hypoglycaemia. Age, partial immunity to malaria, delay in malaria diagnosis and the level of parasitaemia were not associated with death in this cohort.

**Conclusion:**

Severe malaria cases should be continued monitored in the ICUs. SAPS II and the WHO score are good predictors of mortality in malaria patients, but other specific scores deserve to be studied prospectively.

## Background

The number of malarial infections acquired by international travellers, thought to be nearly 25,000 cases annually, remains very small compared with the annual global incidence of nearly 250 million malaria cases and about 700,000 related deaths in endemic areas, mainly among young children in Africa. Malaria in non-endemic countries is a challenge since, due to the fact that it is infrequent, experience in diagnosis and treatment is scarce and delays in diagnosis and adequate treatment may be fatal to the patient [1-3].

The diagnosis of malaria requires a high index of suspicion, as symptoms can mimic many other diseases. Portugal maintains a close relationship with African countries for several reasons and so malaria must be suspected in travellers that return from those countries.

The present study includes expatriates (emigrants), people that were born and live in Africa travelling to Portugal to visit friends and relatives, immigrants (people that were born in an endemic area and that live in Portugal) and those travelling for business or tourism.

Malaria is a notifiable disease in Portugal; about 50 cases are reported annually to the Public Health System, but cases are under-reported [4]. The notification implies filling-in a form that needs to be posted, which leads to forgetfulness. A person from a non-endemic country (emigrant or expatriate) who stays in malaria-affected areas for less than two years is considered non-immune, and also at greater risk of more severe forms of the disease [5]. Non-immune people can develop symptoms of falciparum malaria within a month after leaving the endemic area (average 10 days) but, sometimes, after longer periods as can be observed in pregnant women, immigrants, people that were prescribed mefloquine and HIV-infected people [6-8].

Severe forms of malaria are almost always caused by *Plasmodium falciparum*; though rare, vivax malaria can also cause severe disease. The object of this study is falciparum malaria solely. Severe forms of malaria should be regarded as a medical emergency and managed in intensive care units (ICU) [9-11]. Clinical deterioration usually develops three to seven days after fever onset, but it can sometimes develop in the first 24 hours. A high standard of care and continued monitoring in the acute stage is very important to reduce mortality [12,13].

In 1990, the World Health Organization (WHO) established the criteria for severe malaria which were revised in 2010 (Table 1), with the purpose of identifying individuals at risk of dying and specifying the risk factors of severe forms of the disease. The guidelines for the treatment of malaria published in 2006 were also revised in 2010 [14-16].

**Table 1:** WHO criteria for severe malaria [10,15,17]

Manifestation	Definition
Cerebral malaria	Impaired consciousness or unrousable coma not attributable to any other cause, with a Glasgow score ≤ 9. Prostration, i.e. generalized weakness so that the patient is unable to walk, or sit up without assistance Failure to feed Multiple convulsions - more than two episodes in 24 h

Severe anaemia	Haematocrit < 15% or haemoglobin < 5 g/dl in the presence of parasite count > 10 000/μl

Renal failure	Urine output < 400 ml/24 hours in adults (< 12 ml/kg/24 hours in children) and a serum creatinine > 265 μmol/l (> 3.0 mg/dl) despite adequate volume repletion

Pulmonary oedema and ARDS	The acute lung injury score is calculated on the basis of radiographic densities, severity of hypoxemia, and positive end-expiratory pressure

Hypoglycaemia	Whole blood glucose concentration < 2.2 mmol/l (< 40 mg/dl)

Circulatory collapse	Systolic blood pressure < 70 mmHg in patients > 5 years of age (< 50 mmHg in children aged 1-5), with cold clammy skin or a core-skin temperature difference > 10°C

Abnormal bleeding and/or disseminated intravascular coagulation	Spontaneous bleeding from gums, nose, gastrointestinal tract, or laboratory evidence of disseminated intravascular coagulation

Repeated generalized seizures	≥2 seizures observed within 24 hours

Acidaemia/acidosis	Arterial pH < 7.25 or acidosis (plasma bicarbonate < 15 mmol/l)

Macroscopic haemoglobinuria	Haemolysis not secondary to glucose-6-phospha e dehydrogenase deficiency

Impaired consciousnesss	Rousable mental condition

Prostration or weakness	Generalized weakness so that the patient is unable to walk or sit up without assistance

Hyperparasitaemia	> 2% parasitized erythrocytes or > 250 000 parasites/μl (in non-immune individuals)

Hyperpyrexia	Core body temperature > 40°C

Hyperbilirubinaemia	Total bilirubin > 43 μmol/l (> 2.5 mg/dl)

In general, there is a correlation between the parasite density in the peripheral blood and the severity of the disease and its complications, especially among non-immune people. In such cases, the patients' clinical condition can deteriorate even after initial appropriate treatment due to exacerbation of systemic inflammatory response, leading to organ dysfunction [17-19].

The aim of this retrospective study is to describe the clinical spectrum of severe malaria cases admitted to an ICU and identify factors associated with in-ICU mortality.

## Methods

Between January 1990 and May 2011, 284 patients suffering from malaria were admitted to the Infectious Disease Service of University Hospital in Oporto, northern Portugal. From this cohort, 59 severe malaria cases, according to WHO criteria, were admitted to the ICU of the Infectious Diseases Service (ICU-ID). Patients charts were reviewed retrospectively as well as data on demographic characteristics, epidemiologic history and symptoms of the disease. The study had the approval of the ethics committee of Hospital S João.

Parasitological diagnosis was done by thin smear and light microscopic observation; parasitaemia quantification was done whenever possible. Daily smears were done until the *Plasmodium *smear became negative. Since 2000, a immunochromatographic assay for the qualitative detection of *Plasmodium *antigens were also used at the emergency department (Malaria Now- Binax^®^). Clinical and laboratory parameters were analysed in order to determine which of them were associated with ICU mortality. To evaluate severity at the ICU admission, the Simplified Acute Physiology Score II (SAPS II) was applied.

The degree of immunity to malaria was estimated as follows: a) people who had been living in an endemic area for at least two years (emigrant) at the time of the diagnosis were presumed semi-immune, as well as adult migrant Africans that come to Portugal to visit friends or relatives; b) Europeans who travelled occasionally to endemic areas were considered non- immune. WHO's definition for severe malaria was applied and major and minor indicators considered according to the definitions presented in Table 1. The number of severity criteria was quantified and analysed.

Two prognostic scores of malaria were applied: (1) Malaria Prediction Score (MPS) determined by: 2.13 + 0.02 × (age) + 0.25 × (creatinine) - 0.24 × (haemoglobin) + 3.05 (malaria cerebral criteria) + 0.8 (presence of pregnancy) + 0.8 (ventilated) (where age = age in years; creatinine is in mg/dl, haemoglobin in g/dl; presence of pregnancy, cerebral malaria or ventilatory support, when present = 1, when absent = 0) [20].; (2) Malaria Score for Adults (MSA) was applied to all but three children and the score was determined by: 1 (severe anaemia) + 2 (acute renal failure) + 3 (respiratory distress) + 4 (cerebral malaria). The MSA ranges from 0 to 10 [20].

Variables' definitions were: severe anaemia (Hgb < 5 g/dL), thrombocytopaenia (platelets < than 100 x10^5^/uL); acute respiratory distress syndrome (ARDS) (acute respiratory failure defined by an acute hypoxaemia with the ratio of the partial pressure of oxygen in the patients' arterial blood (PaO_2_) to the fraction of oxygen in the inspired air (FIO_2_) (PaO_2_/FIO_2 _ratio), less than 200 after exclusion of cardiogenic pulmonary oedema by clinical criteria or by a pulmonary capillary wedge pressure (PCWP) < 18 mm Hg in patients with a pulmonary artery (Swan-Ganz catheter); cerebral malaria (unrousable coma), acute renal failure (ARF) (creatinine > 3 mg/dl or urine output < 400 mL/day in adults), liver dysfunction (ALT at least two times the normal value IU/mL), hyperbilirubinaemia (bilirubin value > 2.5 mg/dL), hyponatraemia (Na < 125 mEq/mL), acidaemia (pH < 7.25), and hypoglycaemia (blood glucose < 40 mg/dL).

Community-acquired co-infection was defined as any infection diagnosed within the first two days of hospitalization. Infections occurring later than this were considered nosocomial. ICU admission criteria other than high parasitaemia were any cerebral dysfunction (GCS < 12), haemodynamic instability (SBP < 80 mmHg), respiratory distress (respiratory rate > 20/min or pCO2 < 32 mmHg), jaundice (bilirubine > 2.5 mg/dl) or renal dysfunction (oliguria or creatinine > 3 mg/dl).

Before 1992, the only available treatment for malaria was oral triple therapy (quinine sulphate plus pyrimethamine plus sulphadiazine), whereas after 1992, intravenous quinine dihydrochloride plus clindamycin or doxycycline has been chosen for the treatment of severe cases of malaria. Artemisinin derivatives are not yet available in Portugal. Treatment support includes: blood products to treat severe anaemia and coagulation disorders, intravenous glucose for hypoglycaemia, acetaminophen for hyperpyrexia, if moderate hypoxaemia oxygen by facial mask, antibiotics for bacterial co-infection; haemodynamic and life support according to SSC guidelines since 2005 [21].

Endotracheal intubation and ventilatory support would be done if the patient had respiratory failure or neurologic dysfunction, needing airway protection; if acute oliguric renal failure or severe metabolic acidosis and/or severe electrolytic imbalance were present, renal support, usually a continuous technique, would be started.

Statistical analysis was performed with Stata V12. Descriptive statistics included frequency analysis (percentages) for categorical variables and median and interquartile ranges (IQR) for continuous variables. The differences in characteristics according to the outcome were tested using Fisher exact tests or chi-squared test for categorical variables and Wilcoxon tests for continuous variables.

## Results

### Epidemiology

From January 1990 to May 2011, a total of 284 patients suffering from malaria were admitted. Fifty nine (20.7%) patients were within the criteria of severe malaria and were admitted in the ICU-ID, a level III medical-surgical unit. Three patients were children aged one, nine and 11. Adults' ages varied from 20 to 71, with a median value (IQR) of 42 (33-50), and six were older than 60; 47 (79.6%) were male.

Forty one (69%) were Portuguese emigrants working in Africa (for a period of two or more years in 20 patients and varying from two to 34 years; for a period of up to two years in14 patients and for a period that has not been defined in 7); seven patients were tourists and other seven lived in Africa and came to Portugal (five were Africans that came to visit friends and relatives and two were immigrants), and four worked in boats that had made several stops in seaports along the way. All but three had returned from sub-Saharan Africa: 48 (81.3%) from former Portuguese colonies in Africa - Angola (31), Mozambique (9), Guinea-Bissau (7) and Sao Tome and Principe (1); the others had returned from Senegal (2) and Gabon, Malawi, Burkina Faso, Central African Republic, Nigeria, and Côte d'Ivoire, one patient from each country; two other patients had returned from Thailand and one from the Philippines. Fifty one patients (86.4%) were white, six black and two Asian. Malaria immune status was evaluated in 54 (91.5%) patients: 25 (46.3%) were non-immune and 29 (53.7%) semi-immune, according to the defined criteria. Chemoprophylaxis was prescribed to 14 (23.7%) patients, but discontinued or not taken properly, except from one patient with cerebral malaria that was on mefloquine chemoprophylaxis.

Co-morbidities were present in 15 (25.4%) patients: alcoholic hepatic disease, diabetes mellitus and ischemic cardiac disease in five patients; sickle cell anaemia, nephrolithiasis, Still disease and hepatitis C were observed in one patient each. One had been bone marrow transplanted seven years before and was asymptomatic, with no immunomodulatory therapy. An HIV primary infection was diagnosed simultaneously with malaria in one patient, being all other patients HIV negative. Some patients presented more than one co-morbidity.

### Clinical features

The diagnosis was made between the day of return from the endemic area up to the 47th day in 54 patients; in one patient, diagnosis was done on the 120th day after leaving Angola, where he had been for 16 consecutive months; in other four patients, the gap was impossible to determine.

Symptoms, namely fever before admission, varied from one day to three weeks, the median (IQR) duration being more than seven days in 44% of the patients. All patients had fever. Other complaints include chills in 49 (83.0%) patients, headache in 47 (79.6%), myalgia in 45 (76.2%), vomiting in 27 (45.7%), diarrhoea in 21 (35.6%) and cough in 13 (22.0%); clinical deterioration with cerebral dysfunction (Glasgow ≤9) in 22 (37.2%) patients and respiratory distress in 22 (37.2%) patients were observed.

SAPS II was registered in 56 patients and the median value (IQR) was 33 (24-46). Malaria diagnosis was established by positive smears for asexual *P. falciparum *forms. In 48 (81.3%) patients, parasitaemia quantification was ≥ 2% and varied from 2-90%, with a median (IQR) of 15 (9-30). Parasitaemia was considered high but was not quantified in 13 (22%) patients. The immunochromatographic assay in whole blood for the qualitative detection of *Plasmodium *antigens, used at emergency department, was positive in all the samples tested.

The most common analytic changes observed in these patients were: thrombocytopaenia in 54 (91.5%) patients associated with DIC in seven (11.8%) and bleeding disorders in another six, hyponatraemia in 34 (57.6%) patients, acute renal failure in 31 (52.5%) patients, being oliguric in 18 (30.5%) of them and liver dysfunction in 27 (45.7%) patients. None of the patients presented severe anaemia according to the WHO criteria but, despite transfusions received, 14 patients had haemoglobin less than 8 g/dL with the minimum value of 5.1 g/dL, being the haemoglobin IQR of 10.1 (8.0-12.6).

**Figure 1:**
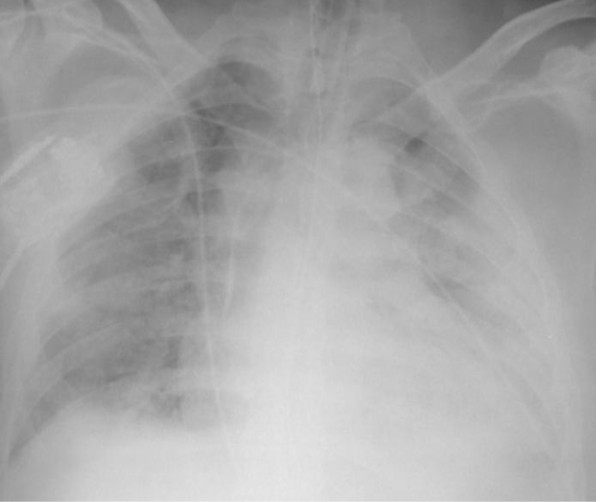
**X-ray: bilateral pulmonary infiltrates (ARDS)**.

**Figure 2:**
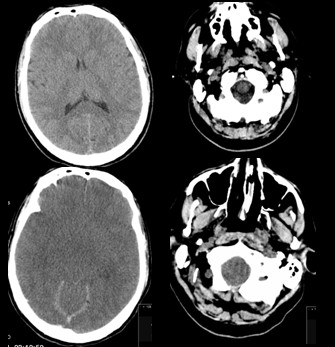
**CT- scan: cerebral swelling in intracranial hypertension (Cerebral malaria)**.

The criteria for severe malaria were: hyperparasitaemia in 46 (77.9%) patients, renal failure in 31 (52.5%), shock in 29 (49.1%), hyperbilirrubinaemia in 27 (45.7%), acidaemia in 23 (38.9%), pulmonary oedema or ARDS in 22 (37.2%) patients (Figure 1), cerebral dysfunction in 22 (37.2%), 11 of whom in unresponsive coma (Figure 2), hypoglycaemia in 18 (30.5%), and haemorrhagic events or DIC in 13 (22.0%) patients.

Considering the major WHO criteria of severe malaria, 29 (49.1%) patients presented five or more dysfunctions.

### Anti-malarial treatment

Forty one (69.5%) patients were treated with quinine dihydrochloride, with a 20 mg/Kg loading dose followed by 10 mg/Kg tid, in a four-hour infusion associated with endovenous clindamycine. The other 18 patients (30.5%) were treated with oral quinine sulphate associated with doxycycline or pyrimethamine plus sulphadiazine in the first period of the study. Blood smears became negative for *Plasmodium *from the second to eighth day of treatment, except in a patient whose parasitaemia was 90% and who died with positive smears in the second day after admission.

### Critical care management

All patients admitted to the ICU were close monitored and had treatment support according to the dysfunctions presented, namely transfusions of packed red cells whenever haemoglobin was lower than 7.5 g/dL, associated with platelets and fresh frozen plasma in patients with bleeding disorders; blood products were also given before an invasive technique whenever platelets were lower than 50 × 10^5^/uL or if coagulopathy was present. Fluids were administered to correct hypovolaemia monitored by central venous pressure, mean arterial pressure and urinary output. Even so, 29 (49.1%) patients needed vasopressor support, with noradrenaline or dopamine or both. Ventilatory support was needed in 22 (37.3%) patients, for a period varying from two to 74 days (median 10 days). Two patients with severe ARDS with refractory hypoxaemia had been put on venovenous Extracorporeal Membrane Oxygenation (ECMO). In one of the patients, ECMO was completely successful and the patient is now doing well, without significant respiratory sequels; the other patient died on ICU in refractory shock. Eight (13.6%) patients required renal replacement therapy. Six patients had upper gastrointestinal bleeding and did an endoscopy for further study. All patients began enteric nutrition in the first 48 hours, if not contraindicated. Glycaemia control was monitored, being hypoglycaemia registered and treated in 18 patients (30.5%).

### Bacterial co-infections

Other simultaneous infections occurred in some patients. Thirteen (22.0%) patients had pneumonia: in two of them, pneumonia was considered community-acquired; *Pneumococcus *and *Klebsiella *spp. were isolated from the tracheal aspirate, in each patient. In the remaining 11 cases, pneumonia was nosocomial and, apart from one isolate (an MDR *Pseudomonas aeruginosa*), no agent was identified; six of these patients were ventilator associated pneumonia. All patients but one recovered.

Another seven (11.9%) patients developed acute acalcoulous cholecystitis, which was managed with medical treatment in five and, in the other two, a percutaneous drainage was done, with a favourable evolution in all of them. Two (3.3%) patients had nosocomial central venous catheter-associated sepsis, having *Acinetobacter baumanni *been identified (from central catheter and peripheral venipuncture) in one patient and *Pseudomonas aeruginosa *MDR in the other; both were treated with antibiotics and had a favourable outcome.

### Outcome

The UCI stay ranged from one to 81 days, being the average (IQR) four days (two to twelve) in survivors and eight days (three to twenty seven) in the patients that died (p = 0.185). All patients who came out alive from the ICU were discharged from the hospital.

In two of them, an acute disseminated encephalomyelitis (ADEM) was diagnosed based on neurologic dysfunction and cerebral resonance magnetic image; both had a favourable outcome. All patients were also evaluated after hospital discharge for follow-up of residual dysfunctions and no other sequelae were detected; in those whose rapid malaria test had been consistent with coinfection with *P vivax*, primaquine was prescribed after excluding Glucose-6-Phosphate Dehydrogenase deficiency.

The global case fatality rate of the infectious diseases department was 3.1%, but the ICU fatality rate was 15.2% (nine patients). Eight were workers: seven returned from Angola, one from Thailand and the 9th was a tourist who returned from Mozambique. Death occurred between the second and 28th day. In all but three patients death occurred in the acute phase of the illness. One of them was a male nurse who, despite compliance to mefloquine chemoprophylaxis, had malaria with 30% of parasitaemia; he was conscious when he was admitted but had a rapid neurologic deterioration in some hours with severe intracranial hypertension and an irreversible brain swelling on cerebral computerized tomography image, with brain death in the first 24 hours. In the other three patients, who died later on, death was related to ARDS complications.

### Statistical analysis

The variables associated with death (Table 2) were SAPS II (average: 68 *vs*.29; p < 0.001), cerebral malaria (44.4% *vs *14.6%; p = 0.019), acute renal failure (88.9-% *vs *46.0%; p = 0.027), respiratory failure (88.9% *vs *29.2%; p < 0.001), hypoglycaemia (77.8% *vs *22.4%; p = 0.002), DIC or spontaneous bleeding (62.5% *vs *13.6%; p = 0.007), and acidaemia or acidosis (100% *vs *29.2%; p < 0.001). Shock (77.8 vs 44.9; p = 0.08) and hyperbilirubinaemia (77.8% vs 40.4%; p = 0.066), although more frequent in patients who died, did not reach statistical significance. The duration of the symptoms before admission has no statistical significance when considering the risk of death (p = 0.82), but the same was not true when we analysed the time since the patients' arrival from the endemic area to diagnosis, which was statistically associated with a worse prognosis (p = 0.02).

**Table 2:** Comparison of different parameters between severe malaria cases who survived and those who died in ICU

	Survivors (n = 50)	Non-survivors (n = 9)	P*
Age [median IQR]	42 (33-49)	45 (33-57)	0,353

Male gender [n (%)]	38 (76.0)	9 (100)	0.161

Duration of symptoms [median IQR]	7 (4-9)	6 (4-8)	0.828

Time since arrival to diagnosis [median IQR]	8 (6-15)	16 (10-20)	0.027

Non-immune (*vs *semi-immune) status [n (%)]	24/46(52.2)	5/8 (62.5)	0.711

Parasitaemia [n (%)]	15 (30.3)	2 (22.2)	0.252
≤10	19 (38.0)	3 (33.3)	
11-50	4 (8.0)	3 (33.3)	
> 50	12 (24.4)	1 (11.1)	
Non quantified			

SAPS II [median (IQR)]	28.5 (21.5-37)	68 (47-73)	< 0.001

Glasgow coma scale [n (%)] ≥13	33 (68.8)	(22.2)	0.019
10-12 (rousable coma)	8 (16.7)	3 (33.3)	
≤ 9 (unrousable coma)	7 (14.6)	4 (44.4)	

Haemoglobin (mg/dL) [median (IQR)]	9.5 (8.0-12.6)	10.4 (8.3-12.2)	0.618

Renal failure [n (%)]	23 (46.0)	8 (88.9)	0.027

Pulmonary oedema/ARDS [n (%)]	14 (29.2)	8 (88.9)	< 0.001

Hypoglycaemia [n (%)]	11 (22.4)	7 (77.8)	0.002

Circulatory collapse, shock [n (%)]	22 (44.9)	7 (77.8)	0.080

Abnormal bleeding/DIC [n (%)]	6 (13.6)	5 (62.5)	0.007

Acidaemia/acidosis [n (%)]	14 (29.8)	9 (100)	< 0.001

WHO major criteria of severe malaria ≥ 5 [n (%)]	20 (40.0)	9 (100)	< 0.001

Malaria Prediction Score (MPS)^ref ^[median (IQR)]	1.78 (0.38-4.53)	4.68 (4.21-5.20)	0.008

Malaria Score for Adults (MAS)^ref ^[median (IQR)]	3 (1-7)	9 (6-10)	0.001

Length of stay in ICU [median (IQR)]	4 (2-12)	8 (3-27)	0.185

* χ^2 ^test or Fisher exact test for categorical variables, Wilcoxon test for continuous variables. IQR = interquartile ranges

The number of organ dysfunctions as described in septic shock was correlated with prognosis and this is well documented by the presence of more than four WHO major criteria of severe *falciparum *malaria and the risk of death (p < 0.001). Renal replacement (88.9% *vs *46.0%; p = 0.027) and mechanical ventilation (88.8% *vs *28.0%; p < 0.001) were also significantly more frequent in those patients who died. The median (IQR) duration of the ICU stay increased, but without reaching statistical significance in the nine patients who died (3-27) *vs *4 (2-13); p = 0.575).

**Figure 3:**
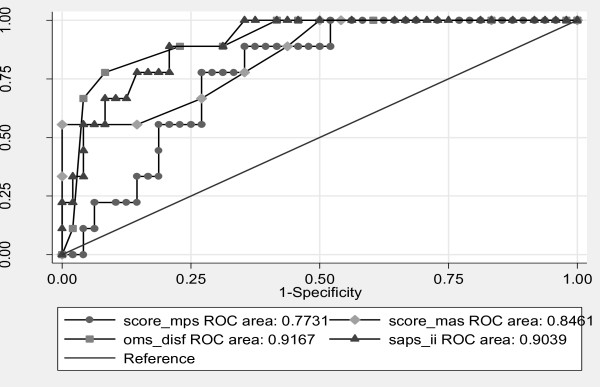
**ROC curve of four scores death predictors**.

The MPS ranged from 1.78-5.20 (median, IQR: 1.8; 0.31-3.0) and the MSA from 3-9 (median; IQR: 2; 0-5). Comparing the median value of the two prognostic scores, both were good predictors of death in this cohort (Table 2). When the four scores were compared, MPS (AUC 0.77; IC95% 0.64-0.90), MSA (0.84; IC95% 0.70-0.98), SAPS II (0.90; IC95% 0.81-0.99) and WHO score (0.91; IC95%0.82-1.0), based on the area under the ROC curve, the best death predictors were SAPS II and the WHO scores (Figure 3).

## Discussion

In this retrospective study 59 consecutive patients with severe malaria admitted in a Portuguese infectious disease UCI over a 22 year period were considered. Obviously, the longer the period, the greater the impact of lost data, but the sample did not change over time and it includes mostly adult white males, middle-aged, travelling for work reasons. Malaria is a complex disease and it is well known that the higher the parasitaemia the greater the systemic inflammatory response and the production of cytokines and so, a major risk of organ dysfunctions, but some aspects concerning severe forms need to be clarified [21-23]. The WHO has redefined the criteria of severe malaria and pointed out a list of complications, but prognostic importance of each complication has not been well established. So, parameters usually associated with poor prognosis should not be forgotten and the patients' monitoring according to their presence or possibility, including the admission to an ICU [22].

In this paper, the ICU admission was based on the presence of at least a life-threatening organ dysfunction, haemodynamic instability, as well as high parasitaemia, which often precedes clinical deterioration. Delay in diagnosis of malaria has been associated with a worse prognosis [24,25], but this was not found in this review, where 49% of the patients had the symptoms for more than a week before diagnosis, without any correlation with a worse prognosis. It is difficult to explain the reasons why. The validity of the variable (duration of symptoms) may be questioned because the time since arrival to diagnosis was statistically significant for the risk of death. A delay in diagnosis can be caused by patient ignorance about the potential severity of malaria, auto medication for fever with antipyretics before seeking medical attention or the doctor's delay in considering the diagnosis, only overcome with proper training and experience. In this review, the case fatality rate was 15.2% (nine patients) and factors statistically associated with death were time since arrival to diagnosis (p = 0.027), SAPS II score (p < 0.001), cerebral malaria (p = 0.019), acute renal failure (p < 0.001), ARDS (p < 0.001), hypoglycaemia (p = 0.02), DIC or spontaneous bleeding (p = 0.007) and acidaemia (p < 0.001) (Table 2); the need for renal replacement (p = 0.027) and invasive ventilation (p = 0.027) was significantly more frequent in those who died. These data are in agreement with literature review, where the fatality rate is higher in cerebral malaria and acute respiratory distress syndrome (ARDS). Two of the patients were put on ECMO, used as a rescue therapy [26-28]. They had been working in Angola and Mozambique for some years, had parasitaemia 2% and 3% respectively and severe ARDS and multiorganic dysfunction. One of them survived and recovered without respiratory sequelae, the other died in refractory shock. These cases show the importance of a clinical surveillance of all patients, because the evolution is sometimes unfavourable in cases that initially seemed benign. The patient on mefloquine chemoprophylaxis that died had a parasitaemia of 30% and a fulminant evolution. Unfortunately he was not autopsied and it was impossible to investigate if it was a problem of resistance to mefloquine.

The number of organ dysfunctions may be directly correlated with mortality as was observed in this cohort, where the presence of more than four WHO major criteria for severe *falciparum *malaria significantly increased the risk of death (100% *vs *40%; p < 0.001) [11,29,30].

Unexpectedly, age, duration of symptoms, immune status, parasitaemia level, bilirubin value and the presence of shock were not statistically associated with mortality, even though hyperbilirubinaemia and shock were more frequent in patients who died, but diverse results are found in different studies [31]. These results should be interpreted with some caution because the study covers a small number of patients over a long period of time, although this does not necessarily explain the discrepancies between this and other studies. Previous papers reporting malaria parameters associated with mortality, mainly retrospective, have different aims and different degrees of severity and only a few of them take ICU cohorts into consideration [12,13,32,33]. This is the case of a study reported in 2003 from a French group that evaluated 188 ICU patients with imported *falciparum *malaria over a 12-year period [10] with an overall mortality rate of 5.3% but, in most severe patients, the mortality rate was 11%. Factors associated with death were SAPS II, shock, acidosis, coma, pulmonary oedema and coagulation disorders.

No predictive malaria score system had been used as routine. Two malaria prognostic scores (MSA and MPS) used in this cohort were good death predictors [20]. When the four scores applied, SAPS II, MPS, MSA and WHO score were compared, SAPS II and the WHO score were the most sensitive to predict death (Figure 1).

Patients with severe forms of malaria are highly susceptible to bacterial infections and WHO recommends concomitant antibiotic therapy. In this review, some infections were documented: 13 patients had pneumonia, two of them community-acquired and 11 nosocomial, seven had acute acalculous cholecystitis and, in another two patients, central venous catheter-associated sepsis occurred. Although malaria acute acalculous cholecystitis is rarely reported, seven cases (11.9%) were found in this cohort [34,35].

The HIV primary infection was considered as the patient has been tested six months before, when he was treated for a cellulitis, having on that date a negative test for the HIV virus. The patient had a favourable evolution without concomitant antiretroviral therapy. There is no consensus about severity or complicated malaria in HIV co-infected adults but the fact that fever is a common symptom to both diseases can delay both diagnoses [7].

Post-malaria neurological syndrome, another rare complication, was reported in two patients who recovered completely. This neurological syndrome has been defined as the acute neuropsychiatric manifestation in patients recently recovered from malaria [36].

The prevention of malaria is essential with easy interventions such as different barrier methods and chemoprophylaxis whenever justified. Pre-travel advice and general practitians should stress prevention measures. Although the study does not point out that a delay in diagnosis is directly connected with a worsening of malaria, people at risk should be informed about the malaria symptoms, allowing an early diagnosis and treatment.

Although anti-malarial drug medication is the only intervention of proven efficacy to treat severe malaria, it is very important to monitor and treat severe forms with organic dysfunctions in ICU [37].

## Competing interests

The authors declare that they have no competing interests.

## Authors' contributions

LS, CA designed the study, took part in the clinical management of the patients and wrote the manuscript. SX collected, analysed and prepared the data. RL and MT performed the statistical analysis and contributed to the interpretation of the data. AS took part in the clinical management of the patients and reviewed the manuscript. This study was not supported by any research fund. All authors read and approved the final manuscript.
